# Clinical predictors of malignancy in lymphadenopathy: A multivariable analysis from a quick diagnosis unit

**DOI:** 10.1016/j.clinme.2026.100567

**Published:** 2026-03-16

**Authors:** Eloi Garcia-Vives, Jaime Rodriguez-Morera, Ariadna Brase Arnau, Abora Sergio Rial Villavecchia, Carme Gimenez Argente, Jose Maria Mora Lujan, Mariona Llaberia Torrelles, Jade Soldado Folgado, Maria Lourdes Cos, Irene Petit Salas, Isabel Campodarve Botet, Xavier Nogués Solan

**Affiliations:** a Internal Medicine Department, Hospital del Mar, Passeig Marítim de la Barceloneta n°25-29, Barcelona, Spain; b Medicine Department, Pompeu-Fabra University, Barcelona, Spain; c Hospital del Mar Research Institute, Biomedical Research Networking on Frailty and Healthy Aging (CIBERFES), Barcelona 08003, Spain

**Keywords:** Lymphadenopaties, Lymph node, Quick diagnosis unit, Risk factor, Cost saving, Healthcare system delay

## Abstract

**Background:**

Peripheral lymphadenopathy (LA) has diverse causes and may indicate malignancy, particularly in referred patients.

**Aim:**

To characterise patients referred for unexplained LA to a quick diagnosis unit, and identify independent predictors of malignancy.

**Design and methods:**

We conducted a retrospective study of 485 consecutive adults evaluated for unexplained LA between 2017 and 2023. The primary outcome was malignancy. Secondary outcomes included diagnostic delay and time to oncology referral. Demographic, clinical and laboratory variables were compared across aetiological groups. A parsimonious multivariable logistic regression model included five clinically relevant predictors identified in univariable analyses and supported by biological plausibility.

**Results:**

Median age was 46 years, and time to first visit was 11 days. Cervical nodes were most frequent (51.9%), followed by supraclavicular (18.6%). Malignancy was diagnosed in 20.8% of patients, with diagnostic delay of 26.5 days (15.5–42). Other specific diagnoses were established in 35.5% of cases, while 43.7% were reactive. Malignant cases were older (60.8 vs 42 years), predominantly male (68.3% vs 44.5%), had higher drug exposure (50.0% vs 29.8%), and shorter symptom duration (45 vs 90 days). In multivariable analysis, independent predictors of malignancy were: age (odds ratio (OR) 1.71 per 10-year increase), male sex (OR 3.25), lymph node size (OR 1.36 per 5 mm increase), indurated consistency (OR 3.42), and supraclavicular location (OR 4.96). Median time to oncology evaluation was 47 days.

**Conclusion:**

The QDU model enables timely diagnosis and detects malignancy in over 20% of cases. Recognising clinical predictors may help prioritise high-risk patients and streamline diagnostic pathways.

## Highlights

• A quick diagnosis unit evaluated 485 patients with unexplained lymphadenopathy, diagnosing malignancy in 21% of cases.
• Age ≥50, male sex, node >25.5 mm, induration, and supraclavicular location predicted malignancy.
• QDU enabled rapid diagnosis: 11 days to first visit and 26.5 days to histological diagnosis.
• Ultrasound performed excellently, with 99% sensitivity and negative predictive value.
• Absence of high-risk clinical/ultrasound features identified very low malignancy risk, supporting selective investigation.

## Graphical abstract

**Figure:**
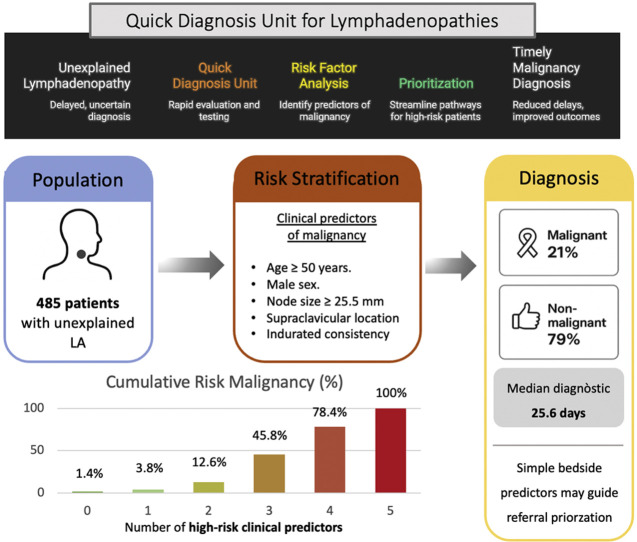

Peripheral lymphadenopathy (LA) encompasses a broad differential diagnosis and may signal underlying solid or haematologic malignancy.^1^ Although it may present with systemic or local symptoms, LA can also as an isolated finding in early stages, making it difficult to distinguish clinically significant conditions from benign or self-limited causes.

The aetiologies of LA include malignant, infectious, autoimmune, iatrogenic and other conditions. Although its prevalence in primary care is low (approximately 0.6%),^2^ the diagnostic workup of LA consumes substantial healthcare resources. The risk of malignancy is estimated at around 1.1% in primary care settings, increases to 11% in referred populations,^1^^,^^2^ and may reach 58% in selected tertiary-care series.^3^

Despite this clinical relevance, there is limited evidence regarding structured outpatient pathways specifically designed to optimise the evaluation of unexplained LA.

To address diagnostic delays and optimise resource use, a dedicated quick diagnosis unit (QDU) for LA was implemented at our tertiary-care centre, integrating internal medicine, radiology and pathology services. Hospital del Mar serves a large urban population with diverse demographic characteristics, distinguishing it from other hospitals in the city.^4^

The primary objective of this study was to determine the proportion of malignant diagnoses among patients referred for unexplained LA to a dedicated QDU. Secondary objectives were to describe the clinical and epidemiological features of this population and to identify independent predictors of malignancy.

## Material and methods

This was an observational, retrospective, single-centre study. Adult patients (≥18 years) who were consecutively referred to the QDU of a tertiary centre between January 2017 and December 2023 were included in the analysis. This study adhered to the STROBE guidelines for the design and reporting of observational research.

Inclusion criteria were the presence of enlarged lymph nodes of unknown cause, evidenced clinically or as an incidental finding in complementary imaging tests requested for another reason (the patient inclusion process is illustrated in Supplementary Fig. S4). The following data were collected retrospectively using electronic patient records: (1) demographic and epidemiological characteristics, (2) clinical and laboratory findings, and (3) timing variables (see supplementary methodology for further details).

Categorical variables were expressed as percentages, and continuous variables by mean ± standard error of the mean (SEM) or median (IQR), according to their distribution. Comparisons between groups were performed using chi-square, Student’s t-test or ANOVA, as appropriate. Welch’s test was used when variances differences were detected using Levene’s test, and non-parametric tests were applied when assumptions were not met. Receiver operating characteristic (ROC) curve analyses were performed to evaluate the discriminatory capacity, with optimal cut-offs determined using the Youden index. For lymph node size, the ROC curve showed good discriminatory ability (AUC = 0.76), and the optimal cut-off identified was 25.5 mm (*see* supplementary methodology for further details). For multivariable analyses, given the limited number of malignant events (*n* = 101), a parsimonious multivariable logistic regression model was constructed, including relevant predictors that demonstrated statistical association in the univariable analyses and were supported by biological plausibility and previous literature. Age and lymph node size were modelled as continuous variables and expressed as odds ratios per 10 year and 5 mm increments. The events-per-variable ratio was approximately 20. The odds ratios (ORs) and their corresponding 95% confidence intervals (CIs) were calculated. For pragmatic clinical interpretation, a cumulative risk analysis was performed by summing the presence of predefined high-risk clinical features (age ≥50 years, lymph node size >25.5 mm, male sex, supraclavicular location, and indurated consistency). The observed proportion of malignancy was calculated according to the number of risk factors present.

Statistical analysis was performed using SPSS v.21.0 software (IBM Corp.). Missing data were handled using complete-case analysis (see supplementary methodology for further details).

## Results

### General and demographic features

A total of 536 patients were referred to a QDU for LA between 2017 and 2023. After excluding 16 patients with incomplete evaluation or loss of follow-up and 35 who did not attend the initial appointment, 485 patients (50.5% women) were included in the analysis, with a median age of 46 (32–62) years. The main demographic, clinical and laboratory features stratified by malignant and non-malignant aetiology are summarised in Table 1. A history of toxic drug use was recorded in 164 cases, and 42/485 (8.7%) had a prior diagnosis of cancer.

**Table 1:** Clinical characteristics of patients with unexplained lymphadenopathy stratified by malignant vs non-malignant etiology.

Factor	Overall (N = 485) n/N (%)	Malignant LA (N = 101) n/N (%)	Non-malignant LA (N = 384) n/N (%)	p
*Epidemiology*				
Age (*years*)	46 (32 – 62)	60.7 ± 1.6	42 (30 – 57)	**<0.001**
Age ≥50 years	-	80/101 (79.2%)	133/384 (34.6%)	**<0.001**
Gender *(men)*	245/485 (50.5%)	69/101 (68.3%)	171/384 (44.5%)	**<0.001**
Ethnicity				
• Caucasian	344/485 (71.1%)	86/101 (85.1%)	258/384 (67.2%)	**<0.001**
• Hispanic	56/485 (11.6%)	7/101 (6.9%)	49/384 (12.8%)	0.103
• Middle Eastern	28/485 (5.8%)	2/101 (2.0%)	26/384 (6.8%)	0.066
• African	13/483 (2.7%)	3/101 (3.0%)	10/384 (2.6%)	0.839
• Asian	43/485 (8.9%)	2/101 (2.0%)	41/384 (10.7%)	**0.006**
Duration (*days*)	65 (30-180)	45 (26 – 90)	90 (30 – 180)	**<0.001**
Duration >100 days	-	17/97 (17.5%)	150/352 (42.6%)	**<0.001**
Drug consumption history	164/482 (34.0%)	50/100 (50.0%)	114/382 (29.8%)	**<0.001**
Oncological history	42/481 (8.7%)	13/101 (12.9%)	29/380 (7.6%)	0.097
Risk factor	101/474 (21.3%)	12/97 (11.9%)	89/377 (23.6%)	**0.016**
Sexual risk relation history	12/482 (2.5%)	0	12/381 (3.1%)	
STD history	13/482 (2.7%)	1/101 (1.0%)	12/381 (3.1%)	0.234
Recent immunization	24/481 (5.0%)	1/101 (1.0%)	23/380 (6.1%)	**0.038**
*Features*				
Indurated	119/457 (26.0%)	61/91 (67.0%)	58/366 (12.8%)	**<0.001**
Attached	82/457 (17.9%)	48/90 (53.3%)	34/367 (9.3%)	**<0.001**
Size >25.5mm	20 (10 – 30)	68/98 (69.4%)	83/352 (23.6%)	**<0.001**
Fever	23/483 (4.8%)	4/101 (4.0%)	19/382 (5.0%)	0.671
B symptoms	12/483 (2.5%)	5/101 (5.0%)	7/382 (1.8%)	0.073
Toxic syndrome	33/483 (6.8%)	20/101 (19.8%)	13/382 (3.4%)	**<0.001**
*Distribution*				
Cervical *(n [%])*	252/485 (51.9%)	60/101 (59.4%)	192/384 (50.0%)	0.092
Supraclavicular	90/485 (18.6%)	38/101 (37.6%)	52/384 (13.5%)	**<0.001**
Submandibular	83/485 (17.1%)	12/101 (11.9%)	71/384 (18.5%)	0.117
Inguinal	87/485 (17.9%)	26/100 (26.0%)	61/384 (15.9%)	**0.019**
Axillary	55/485 (11.3%)	20/101 (19.8%)	35/384 (9.1%)	**0.003**
Mediastinum	28/485 (5.8%)	17/101 (16.8%)	11/384 (2.9%)	**<0.001**
Other	34/485 (7.0%)	16/101 (15.8%)	18/384 (4.7%)	**<0.001**
Extra-nodal	19/485 (3.9%)	18/101 (17.8%)	1/384 (0.3%)	**<0.001**
≥2 non-contiguous territories	37/479 (7.7%)	23/98 (62.2%/23.5%)	14/381 (3.7%)	**<0.001**
*Laboratory*				
LDH (U/L)	179 (155 – 214)	193 (164 – 271)	174 (154 – 205)	**0.011**
β2-microglobulin *(mg/L)*	1.72 (1.43 – 2.18)	2.01 (1.59 – 2.93)	1.61 (1.33 – 2.08)	**<0.001**
Hemoglobin *(g/dL)*	13.9 ± 0.1	13.5 ± 1.2	14.1 ± 0.1	**0.004**
Leukocytes *(x10^3^ cells/µL)*	7.28 (6.24 – 9.09)	9.05 ± 0.46	7.20 (6.04 – 8.65)	**0.003**
Neutrophils *(x10^3^ cells/µL)*	4.10 (3.19 – 5.40)	5.73 ± 0.39	3.95 (3.10 – 4.92)	**0.002**
Lymphocytes *(x10^3^ cells/µL)*	2.27 (1.70 – 2.87)	2.20 ± 0.16	2.37 (1.86 – 2.92)	0.066
ESR *(mm/h)*	16 (6 – 30)	15 (6-38)	17 (6-30)	0.738
One positive haematological tumoral marker*	-	27/52 (55.1%)	15/99 (15.2%)	**<0.001**

The bold values in Table 1 correspond to statistically significant p-values (*p*<0.05).

Categorical variables were expressed as percentages, and continuous variables by mean ± standard error of the mean, or median (IQR), according to their normal distribution. ESR = erythrocyte sedimentation rate; LDH = Lactate dehydrogenase; STD = Sexually transmitted diseases.

⁎ Lactate dehydrogenase > 225 U/L and/or β2-microglobulin > 2.45mg/L. Categorical variables were expressed as percentages, and continuous variables by mean ± standard error of the mean, or median (IQR), according to their normal distribution. STD = Sexually transmitted diseases.

The average time to first consultation was 11 days (5–17), with referral predominantly originating from primary care (79.0%), followed by other hospital departments (11.7%) and emergency department (9.3%).

### LA features and localisation

Median time from LA onset to the consultation was 65 days (30–180), with a size at initial assessment of 20 mm (10–30). In 35 cases, the duration of LA was unknown, as it was discovered incidentally. Medical attention was requested within the first month of onset by 28.7% (139/485 patients; 44.6% women; 48 years (32–63)) of patients.

The most common location was cervical (252/485; 51.9%), followed by supraclavicular (90/485; 18.6%), inguinal (87/485; 17.9%), submandibular (83/485; 17.1%), axillary (55/485; 11.3%), mediastinum (28/485; 5.8%) and other sites (34/485; 7.0%). Extra-nodal involvement was present in 19/485 cases (3.9%), and initial involvement affecting ≥2 non-contiguous regions was noted in 37/485 cases (7.7%).

### Tests performed

Ultrasonography (US) was performed in 386/485 patients (79.6%), showing pathological findings in 53.4%. Sensitivity and negative predictive value (NPV) were 99%, while specificity was 56% and positive predictive value (PPV) was 34%. Computed tomography (CT) scan was performed in 162/485 (33.4%), demonstrating sensitivity of 100% and specificity of 38% (PPV 58%, NPV 100%).

Fine needle aspiration cytology (FNAC) was performed in 146/485 patients (30.1%), core biopsy (CB) in 102/485 (21.0%) and lymph node excision in 52/485 (10.7%).

Among patients with an initial clinical suspicion of malignancy, diagnostic studies confirmed a compatible diagnosis in 71.8% of cases. Conversely, when a benign aetiology was initially considered more likely, malignancy was ultimately diagnosed in only 5.3% of cases.

### Time to and final diagnoses

Ultimately, a diagnosis of malignant LA was confirmed in 101/485 cases (20.8%), with a median time from first visit to histological diagnosis of 26.5 (15.5–42) days. Time from receipt of the initial referral letter to the first consultation in the onco/haematology department was 47 (17–49) days and 82 (34–88) days to treatment initiation.

A non-malignant LA-specific diagnosis was established in 172/485 cases (35.5%), considering non-specific reactive aetiology in the remaining cases (43.7%). The distribution of final diagnoses is detailed in Table 2 (a detailed breakdown of individual diagnosis is provided in Supplementary Table S3). The average number of visits required to establish diagnosis was 1 (1–2).

**Table 2:** Main aetiologies of patients studied in the lymphadenopathy unit.

	*N* = 485
Lymphoid reactive hyperplasia/Unspecific	212/485 (43.7%)
**Malignant disease** *Haematological* • Diffuse large B cell lymphoma • Hodgkin lymphoma • Others^a^ *Oncological* • Head and neck squamous carcinoma • Squamous carcinoma of unknown origin • Others^b^	**101/485 (20.8%)** **64/101 (63.4%)** *21/64 (32.8%)* *14/64 (21.9%)* *29/64 (45.3%)* **37/101 (36.6%)** *15/37 (40.5%)* *5/37 (13.5%)* *17/37 (45.9%)*
**Infections** • Tuberculous lymphadenitis • *Streptococcus pyogenes* tonsillitis • Others^c^	**64/485 (13.2%)** *37/64 (57.8%)* *3/64 (4.7%)* *24/64 (37.5%)*
**Inflammatory** • Post-vaccination • Hashimoto thyroiditis • Others^d^	**32/485 (6.6%)** *9/32 (28.1%)* *5/32 (15.6%)* *18/32 (56.3%)*
**Others** • Branchial cyst • Thyroid nodules • Other^e^	**76/485 (15.7%)** *20/76 (26.3%)* *12/76 (15.8%)* *34/76 (57.9%)*

The bold values in Table 2 correspond to the percentages of the classification groups.

a Included Follicular lymphoma, Chronic lymphocytic leukaemia, Mantle lymphoma, Anaplastic lymphoma, T lymphoma and others.

b Included Thyroid carcinoma, Non-small cell lung cancer, ductal carcinoma breast, ovarian carcinoma, urothelial carcinoma, cutaneous squamous cell carcinoma, oesophageal squamous cell carcinoma, NUT midline carcinoma, GIST, gastric adenocarcinoma, melanoma and others.

c Included HSV-1, EBV, CMV, HIV, parotitis, syphilis, Toxoplasmosis.

d Included Sarcoidosis, Castleman disease, Kikuchi-Fujimoto disease, Interferon treatment and others.

e Included lipoma, parotid tumour, sialolithiasis, fibroma, vascular and others.

Patients with malignant-LA were significantly older (60.7 ± 1.6 vs 42 (30–57); *p* < 0.001; Supplementary Fig. S5), predominantly male (68.3% vs 44.5%; *p* < 0.001) and had greater exposure to drug consumption (50.0% vs 29.8%; *p* < 0.001), as well as shorter duration of LA (45 (26–90) vs 90 (30–180); *p* < 0.001) than those with non-malignant-LA. The differences between the cohorts are summarised in Table 1 and Supplementary Table S4. Supplementary Table S5 reflects the proportion of malignancy according to LA size and location. In univariable analyses (Table 1), age >50, male sex, lymph node size >25.5 mm, indurated consistency, supraclavicular location, shorter symptom duration and other clinical and laboratory variables were significantly associated with malignancy. Variables demonstrating statistical association and clinical relevance were considered for inclusion in the subsequent multivariable modelling.

### Follow-up

Follow-up and serial imaging tests were performed in 149/485 cases, with a median time of 65 (30–180) days. LA enlargement was observed in 5/149 cases (3.4%), stability in 61/149 (40.9%), and size reduction in 83/149 cases (55.7%).

A diagnosis of malignant LA was eventually established in nine cases, with a delay in the imaging tests of 160 days (84.5–210.5). Among these, imaging revealed growth in one case, stability in five and decrease in three. Final diagnoses included lymphoproliferative disorder in seven cases (two diffuse large B cell lymphomas, two chronic lymphocytic leukaemia, one Hodgkin lymphoma, one follicular lymphoma and one undefined lymphoproliferative disorder) and squamous cell carcinoma in the remaining two (one of pulmonary origin and the other unknown primary origin).

### Multivariable analysis of risk factor for malignant diagnosis

**Fig. 1:**
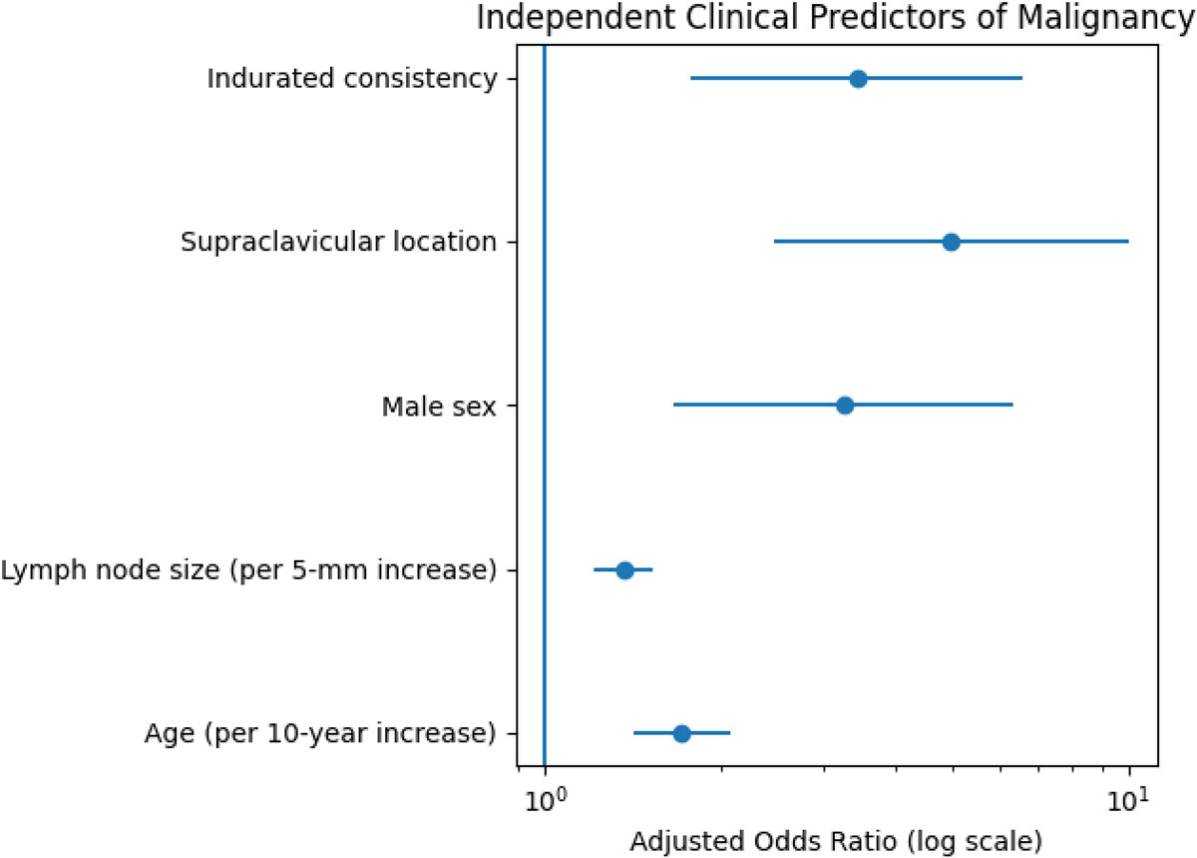
Independent clinical predictors of malignancy.

In multivariable logistic regression model (Supplementary Table S6), increasing age (adjusted OR 1.71 per 10 year increase, 95% CI 1.41–2.07; *p* < 0.001), lymph node size (OR 1.36 per 5 mm increase, 95% CI 1.21–1.53; *p* < 0.001), male sex (OR 3.25, 95% CI 1.66–6.37; *p* = 0.001), supraclavicular location (adjusted OR 4.96, 95% CI 2.46–9.99; *p* < 0.001), and indurated consistency (OR 3.42, 95% CI 1.78–6.60; *p* < 0.001) were independently associated with malignancy. The model demonstrated excellent discrimination (AUC 0.91). The adjusted effect estimates are illustrated in Fig. 1 (full regression coefficients are provided in Supplementary Table S6).

To enhance clinical interpretability, the probability of malignancy increased progressively with the number of risk factors: 1.4% in patients with no risk factors, 3.8% with one, 12.6% with two, 45.8% with three, 78.4% with four, and 100% in those presenting all five features (Table 3).

**Table 3:** Observed probability of malignancy according to the number of high-risk clinical features. Total *N* analysed = 425 patients (complete-case analysis for the five risk factors).

Number of risk factors	*N*	Malignant cases	Malignancy (%)
0 1 2 3 4 5	73 132 103 72 37 8	1 5 13 33 29 8	1.4% 3.8% 12.6% 45.8% 78.4% 100%

High-risk features defined as: age ≥50 years, lymph node size >25.5 mm, male sex, supraclavicular location, and indurated consistency.

## Discussion

This study provides a comprehensive evaluation of an ethnically diverse cohort of 485 patients with enlarged lymph nodes assessed through a QDU. Malignancy was diagnosed in 21% of patients, highlighting the clinical importance of efficient referral and diagnostic pathways. Several key factors – increasing age, larger lymph node size, male sex, indurated consistency and supraclavicular location – were independently associated with malignancy and should be considered in the risk stratification of patients presenting with LA. Age and lymph node size were modelled as continuous variables, demonstrating a progressive increase in malignancy risk with advancing age and nodal size.

The QDU concept was first described in the UK in 1996, under the name of Rapid and Early Diagnosis,^5^ and later redefined by Bosch *et al.* in 2009.^6^ Since then, QDUs have been increasingly implemented as outpatient strategies to improve diagnostic efficiency while reducing unnecessary hospital admissions.^7^^,^^8^, ^9^

In 2006, the Catalan Health System launched the QDU for breast, lung, colorectal, prostate and bladder cancer to reduce the time elapsed between a suspicion, diagnostic and the start of treatment. These pathways aimed to reduce the interval between suspicion and treatment initiation to 30 days.^10^ Although LA was not originally included in this programme, similar outpatient diagnostic pathways have progressively been implemented.

In our cohort, the QDU achieved a mean waiting time of 11 days from referral to first consultation. Although the absence of a direct comparator cohort precludes definitive conclusions regarding delay reduction, this interval compares favourably with publicly reported waiting times in our regional healthcare setting.^11^ Furthermore, 80% of referrals originated from primary care, reducing unnecessary emergency department visits and hospitalisations. While the interval to oncological assessment (47 days) and treatment initiation (82 days) exceeds the 30-day target established for formally funded oncologic fast-track pathways within the Catalan Health System,^10^ previously published data from conventional diagnostic circuits report substantially longer median delays.^11^ In this context, the timelines observed in our cohort compare favourably with historical standards. However, given the observational design and lack of a parallel control group, these findings should be interpreted as descriptive and hypothesis-generating rather than demonstrating superiority. Moreover, several nuances must be considered. The first is that since it is not an officially established pathway, it has neither the priority nor resources assigned to others. Furthermore, the study period included the SARS-CoV-2 pandemic, which may have influenced referral patterns and healthcare accessibility. The precise quantitative impact of COVID-19 on these intervals cannot be determined from the available data. Additionally, many haematological malignancies do not warrant immediate treatment, which also contributes to observed delays.

In cancer, a negative correlation between delayed diagnosis and survival rates appears to be increasingly clear.^12^^,^^13^ In breast cancer, an 8-week delay in initiating treatment increases mortality by 17%, reaching 26% after 12 weeks. Even a 2-week delay can impact outcomes.^13^ Although delays may be multifactorial (stemming from patient, disease or system), there is a clear need to optimise triage and diagnostic circuits, particularly for suspected malignancy. One essential step is refining referral criteria to reduce the gap between demand and available resources, allowing in-depth evaluation to focus on patients with a higher pre-test probability of malignancy.

Our data support this approach. Among the patients diagnosed with malignancy, 75% had presented with LA of less than 3 months’ duration. No malignancies were detected in patients with LA exceeding 12 months, corroborating findings from previous studies suggesting a benign course in persistent, long-standing lymphadenopathy.^14^ The parsimonious multivariable model confirmed that age, nodal size, sex, consistency and anatomical location were independently associated with malignant aetiology, with stable confidence intervals and an adequate events-per-variable ratio. Age and lymph node size were modelled as continuous variables and expressed per clinically meaningful increments (10 years and 5 mm, respectively), allowing more intuitive interpretation of risk. Notably, each 5 mm increase in lymph node size was associated with a 36% increase in the odds of malignancy, reinforcing the progressive nature of risk beyond arbitrary cut-offs.

Importantly, the cumulative risk analysis revealed a marked stepwise increase in malignancy probability according to the number of high-risk features present. Patients without any adverse clinical characteristics had a malignancy rate below 2%, whereas the presence of three or more predictors was associated with a sharp escalation in risk exceeding 45% (Table 3). This pattern supports the practical value of simple bedside risk stratification.

Although derived from a continuous multivariable model, this cumulative approach is intended for pragmatic interpretation and does not replace formal probability estimation.

Previous studies have consistently identified age and lymph node size among the strongest predictors of malignancy.^15^, ^16^, ^17^, ^18^ Similarly, the supraclavicular location and indurated consistency have been repeatedly associated with higher malignant yield.^19^^,^^20^ In our cohort, modelling age and nodal size as continuous variables demonstrated a progressive increase in malignancy risk with advancing age and nodal enlargement. Each 5 mm increase in lymph node size was associated with a 36% increase in the odds of malignancy, supporting the concept that risk increases gradually rather than abruptly at arbitrary cut-offs. Male sex was also associated with increased malignancy risk, potentially reflecting a higher prevalence of toxic habits, as supported by our multivariable analysis and previous studies.^18^^,^^19^

Importantly, when applying pragmatic clinical criteria – age <50 years, lymph node size ≤25.5 mm, absence of supraclavicular involvement, non-indurated consistency, and symptom duration <100 days – only five of the 101 malignant cases (4.9%) were classified as low risk, while 96 (95.1%) clustered in the high-risk category. Although not a validated prediction rule, these criteria may help refine referral strategies. Younger patients with small, soft, non-supraclavicular nodes and longer symptom duration could be considered for short-term observation in primary care, whereas early referral should be prioritised for individuals presenting high-risk features. Prospective validation studies would be required before implementing formal risk-based referral algorithms. These findings align with other proposed diagnostic algorithms^21^ that incorporated easily obtainable clinical variables – such as age, node size, pain, supraclavicular involvement and generalised pruritus – to guide biopsy decisions.^22^ One such model correctly identified 96% of patients requiring biopsy, with a negative predictive value of 98%.^23^

The integration of US as a first-line diagnostic tool further enhances the accuracy and efficiency of QDU pathways. US offers numerous advantages, including accessibility, safety, absence of ionising radiation and high diagnostic yield. In our cohort, the US demonstrated a sensitivity and NPV value of 99%, consistent with other cohorts.^18^ The absence of sonographic features indicative of malignancy – such as enlarged axial diameter, irregular borders, altered echogenicity, and aberrant vascularisation^24^ – was a reliable exclusion criterion, allowing many patients to avoid more invasive procedures. However, a small number of patients who were ultimately diagnosed with malignancy did not show clear radiological progression during follow-up, with some nodes remaining stable or even decreasing in size. These observations caution against over-reliance on imaging stability as a surrogate of benignity and highlights the need for continued clinical vigilance, particularly in patients with persistent risk factors or evolving symptoms. Accordingly, imaging findings should complement – but not replace – longitudinal clinical assessment and individualised risk stratification.

The expanded use of US has also facilitated the increasing use of FNAC and CB, often performed during the same visit. This reduces reliance on excisional biopsy, traditionally considered the diagnostic gold standard but associated with more complications. While FNAC is well established in the diagnosis of metastatic solid tumours, its role in haematological malignancies is more controversial.^22^ Nonetheless, recent advances in immunohistochemical techniques and flow cytometry have substantially improved the diagnostic accuracy of FNAC and CB in this context.^25^^,^^26^ In our series, 72% of patients with haematological malignancy underwent CB, and 44% required excisional biopsy. Only one case was definitively diagnosed through FNAC alone, underscoring the complementary role of multiple diagnostic modalities in these complex cases.

This study is not without limitations. Its retrospective, single-centre design may limit generalisability, and some data loss was unavoidable. Additionally, the study population consisted of referred patients evaluated in a specialised QDU, which may introduce selection bias and limit extrapolation to primary care settings. The study period spanned several years (2017–2023), including the COVID-19 pandemic, which may have influenced referral patterns, healthcare accessibility and diagnostic delays. Although the diagnostic workflow remained structurally unchanged, these temporal factors could have affected case mix and timelines.

Nevertheless, it represents the largest Spanish cohort evaluating LA within a QDU context, offering valuable insights into risk stratification and resource optimisation. Our findings support a more nuanced approach to the management of LA, identifying patients who warrant comprehensive evaluation while avoiding unnecessary investigations in low-risk cases.

## Conclusion

In summary, the QDU model for LA represents a structured outpatient strategy that facilitates timely diagnostic assessment of potentially serious conditions, identifying malignancy in more than 20% of referred patients.

The absence of high-risk features – including younger age, smaller node size, absence of supraclavicular involvement and non-indurated consistency – supports a conservative approach in selected low-risk cases, particularly when LA has persisted for over 3 months.

A simple cumulative assessment of five key clinical features may allow rapid bedside risk stratification and facilitate prioritised referral and diagnostic evaluation.

## Ethics approval and consent to participate

The study was approved by the institutional review board of Hospital del Mar [2023/10724] in accordance with the Declaration of Helsinki. Because of the retrospective nature of the study, informed consent was not required.

## Data availability statement

The datasets generated and/or analysed during the current study are not publicly available due to institutional and ethical restrictions, as they contain sensitive clinical information from patients evaluated at our centre. De-identified data may be made available from the corresponding author upon reasonable request and with permission from the Hospital del Mar Institutional Review Board.

## CRediT authorship contribution statement

**Eloi Garcia-Vives:** Writing – original draft, Methodology, Investigation, Formal analysis, Data curation, Conceptualization. **Jaime Rodriguez-Morera:** Writing – original draft, Data curation. **Ariadna Brase Arnau:** Writing – review & editing, Data curation. **Abora Sergio Rial Villavecchia:** Writing – review & editing. **Carme Gimenez Argente:** Writing – review & editing. **Jose Maria Mora Lujan:** Writing – review & editing. **Mariona Llaberia Torrelles:** Writing – review & editing. **Jade Soldado Folgado:** Writing – review & editing. **Maria Lourdes Cos:** Writing – review & editing. **Irene Petit Salas:** Writing – review & editing. **Isabel Campodarve Botet:** Writing – review & editing. **Xavier Nogués Solan:** Supervision.

## Declaration of competing interest

The authors declare that they have no known competing financial interests or personal relationships that could have appeared to influence the work reported in this paper.

## Appendix Supplementary materials

Image, application 1
