# Supplementary material for: Clinical predictors of malignancy in lymphadenopathy: A multivariable analysis from a quick diagnosis unit
Source: Clin Med (Lond). 2026 Mar 16;26(3):100567. doi: 10.1016/j.clinme.2026.100567 (PMC13096957; doi:10.1016/j.clinme.2026.100567)
Supplement: Supplementary file 1 [file mmc1.docx]

**Supplementary Material**

*Clinical Predictors of Malignancy in Lymphadenopathy: A Multivariable Analysis from a Quick Diagnosis Unit*.

Eloi Garcia-Vives^1^, Jaime Rodriguez-Morera^1^, Ariadna Brase Arnau^1^, Abora Sergio Rial Villavecchia^1^, Carme Gimenez Argente^1^, Jose Maria Mora Lujan^1^, Mariona Llaberia Torrelles^1^, Jade Soldado Folgado^1^, Maria Lourdes Cos Esquius^1^, Irene Petit Sala^1^, Isabel Campodarve Botet^1,2^, Xavier Nogués Solan^1,2,3^.

1.- Internal Medicine Department, Hospital del Mar, Barcelona, Spain.

2.- Medicine Department, Pompeu-Fabra University, Barcelona, Spain.

3.-Hospital del Mar Research Institute, Centro de Investigación Biomédica en Red de Fragilidad y Envejecimiento Saludable (CIBERFES), Barcelona 08003, Spain.

Supplementary Methodology:

- Material and Methods

Patients who were in poor general condition and unable to complete the diagnostic workup in the outpatient setting, as well as those who were lost to follow-up before a final diagnosis was established, were excluded from the analysis. The patient inclusion process is illustrated in Supplementary Figure S4. Referrals came from primary care, Emergency Department and from other departments in the hospital.

For the purpose of this study, “unexplained lymphadenopathy” was defined as lymph node enlargement persistent after prior medical evaluation without an evident diagnosis established by the referring physician. As the most referrals originated from primary care or hospital departments, patients had already undergone an initial clinical assessment aimed at excluding obvious causes.

No minimum size threshold was required for referral, since LA was frequently assessed clinically without standardized imaging measurements.

Patients with clearly identified acute infectious or inflammatory cause at initial evaluation were not referred to a QDU.

Referral triage was primarily determined by the referring physician. All referrals were reviewed prior to acceptance into the QDU to confirm the absence of a clear alternative diagnosis and to avoid duplication of ongoing diagnostic workups.

The collected data included:

1. General information: demographic and epidemiological features, past history of immunization, infection or malignancies, as well as existence of any risk factor for them. History of substance use (alcohol, tobacco or illicit drugs) was also documented.
2. Clinical or laboratory data: Clinical manifestations and disease course were recorded, including LA location, size, consistency (soft, elastic or hard), mobility (mobile or fixed), as well as progression time. “Indurated consistency” was operationally defined as a firm or hard nodal texture on palpation, with reduced compressibility compared to surrounding tissue, as assessed during physical examination by the evaluating physician. Although palpation is inherently subjective, assessments were performed by experienced internists according to routine standardized clinical practice within the unit.

Laboratory parameters included white blood count, hemoglobin, erythrocyte sedimentation rate, lactate dehydrogenase and β2-microglobulin levels. Traditional solid tumour markers were not routinely requested, as they were not considered useful as diagnostic tests, due to the high rate of false positives. Serological studies were requested according to the criteria of the evaluating physician.

The imaging studies-including computed tomography (CT scan), magnetic resonance imaging (MRI) and ultrasonography (US)- and their data were recorded. The need for fine-needle aspiration cytology (FNAC), core-needle biopsy (CB) or lymph node excision was also documented. Ultrasound malignancy criteria included: rounded morphology, transverse axis greater than 10 mm, absence of echogenic hilum and intraganglionic calcification. On CT scan, malignancy was defined by: absence of intravenous contrast uptake and invasion of adjacent structures.

Escalation to invasive diagnostic procedures was based on clinical suspicion of malignancy (e.g., indurated consistency, location, progressive enlargement), persistence on time, suspicious imaging findings, or inconclusive prior investigations. The choice of technique was determined by nodal location, accessibility, and suspected etiology, with multidisciplinary discussion when required.

1. Timing variables: Date and source of referral, time to first consultation, time to histological diagnosis, follow-up and clinical progression were recorded.

Missing data were handled using complete-case analysis. The proportion of missing values was 8.0% for lymph node size and 5.8% for indurated consistency, while no missing data were observed for age, sex, or supraclavicular location. Patterns of missingness were explored descriptively and did not suggest systematic differences according to malignancy status. Given the limited extent of missing data and the retrospective nature of the study, no imputation procedures were performed.

- Statistical analysis

Normal distribution was determined using the Kolmogorov-Smirnov test.

- - ROC curve analysis:

ROC curves were constructed to evaluate quantitative predictors for malignant LA. The optimal cut-off was defined according to the Youden index. Figure S1 shows the ROC curves for quantitative malignant LA risk factors (lymph node size, age and delay to request medical attention). The sensitivity, specificity, positive predictive value and negative predictive value, according to the chosen cut-off are displayed in the supplementary Table S1. These cut-offs were used for descriptive purposes and in the univariable analysis; however, in the multivariable model, age and lymph node size were analysed as continuous variables to preserve statistical power.


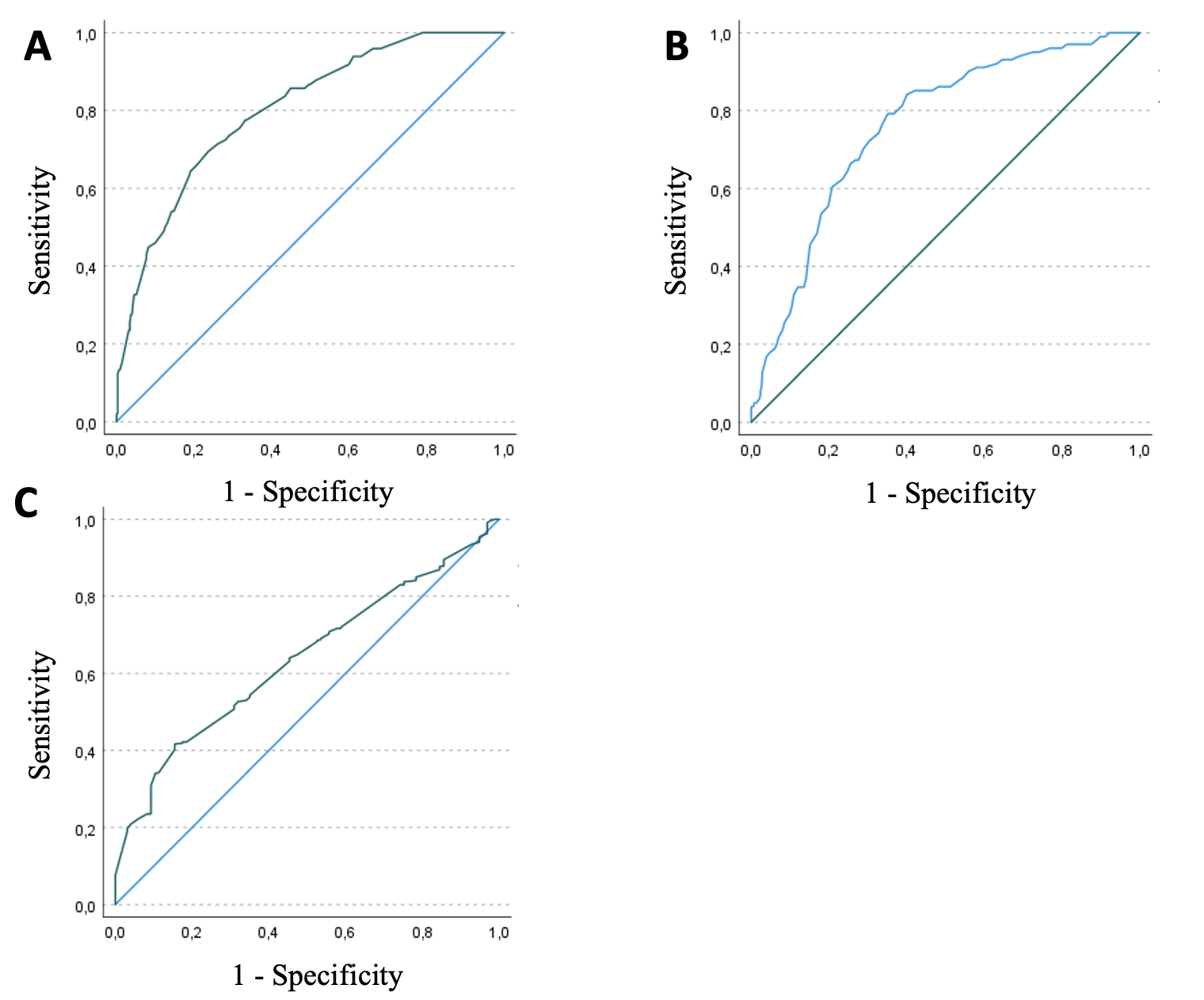


Figure S1: ROC curves analysis for quantitative malignant LA related risk factors, including lymph

node size (A), age at diagnosis (B) and evolution time (C).

| Table S1: Values of sensitivity, specificity, positive predictive value and negative predictive value for the chosen cut-off point of variables related to malignant LA | | | | | | |
| --- | --- | --- | --- | --- | --- | --- |
| **Marker** | **Cut-off** | **AUC** | **Sensitivity (%)** | **Specificity (%)** | **PPV (%)** | **NPV (%)** |
| *Lymph node size*  *Age*  *Evolution* | > 25.5 mm  ≥ 50 years  ≥ 100 days | 0.761  0.802  0.637 | 69.4  79.2  15.5 | 76.4  64.8  58.3 | 45.0  37.2  9.3 | 90.0  92.2  71.5 |
| AUC = Area under curve; NPV = Negative predictive value; PPV = Positive predictive value. | | | | | | |

The majority of quantitative laboratory parameters (white blood count, haemoglobin and erythrocyte sedimentation rate) were not included in the multivariable analysis, as the observed differences were considered to have limited clinical relevance or the Youden-derived cut-off fell within the normal laboratory range, despite statistical significance (Figure S2). In contrast, the differences observed for lactate dehydrogenase and β2-microglobulin in the univariable analysis were considered clinically relevant (Figure S3). As the optimal cut-offs for these variables also lay within the normal range, the upper limit of normality was used as a clinically meaningful threshold, and both parameters were combined into a single variable termed "positive haematological tumour marker" (PhTm) (Table S2).

Figure S2: ROC curves analysis for unselected quantitative malignancy-LA related laboratory risk factors, including leukocytes (A), haemoglobin (B) and erythrocyte sedimentation rate (C).

Figure S3: ROC curves analysis for selected quantitative malignancy-LA related laboratory risk factors, including lactate dehydrogenase (A) and β2-microglobulin (B).

| Table S2: Values of sensitivity, specificity, positive predictive value and negative predictive value for the chosen cut-off point of selected laboratory variables related to malignant LA and normal laboratory range | | | | | | | |
| --- | --- | --- | --- | --- | --- | --- | --- |
| **Marker** | **Normal range** | **Cut-off** | **AUC** | **Sensitivity (%)** | **Specificity (%)** | **PPV (%)** | **NPV (%)** |
| *LDH (U/L)*  *β2-microglobulin (mg/L)*  *PhTm* | *135 – 225  *0 – 2.45  * | > 182  > 1.91  LDH > 225U/L and/or  β2-m > 2.45 mg/L | 0.629  0.684  - | 64.1  56.1  53.8 | 59.7  70.0  86.6 | 39.4  48.9  68.6 | 80.3  78.8  77.4 |
| AUC = Area under curve; LDH = lactate dehydrogenase; NPV = Negative predictive value; PPV = Positive predictive value, PhTm = positive haematological tumour marker, β2-m = β2-microglobulin. | | | | | | | |

*Table S3: Main etiologies of patients studied in the lymphadenopathy unit.*

|  | **N=485** |
| --- | --- |
| **Lymphoid reactive hyperplasia/Unspecific** | **212 (43.7%)** |
| **Malignant disease**  *Hematological*   - Diffuse Large B cell lymphoma - Hodgkin lymphoma - Follicular lymphoma - Chronic lymphocytic leukemia - Mantle lymphoma - Anaplastic lymphoma - T lymphoma - Others   *Oncological*   - Head and neck squamous carcinoma - Squamous carcinoma of unknown origin - Thyroid carcinoma - Non-small cell lung carcinoma - Ductal carcinoma breast - Ovarian carcinoma - Urothelial carcinoma - Cutaneous squamous cell carcinoma - Esophageal squamous cell carcinoma - NUT midline carcinoma - GIST - Gastric adenocarcinoma - Melanoma - Not studied | **101 (20.8%)**  **64 (63.4%)**  *21 (32.8%)*  *14 (21.9%)*  *13 (20.3%)*  *4 (6.3%)*  *4 (6.3%)*  *2 (3.1%)*  *2 (3.1%)*  *4 (6.3%)*  **37 (36.6%)**  *15 (40.5%)*  *5 (13.5%)*  *3 (8.1%)*  *3 (8.1%)*  *1 (2.7%)*  *1 (2.7%)*  *2 (2.7%)*  *1 (2.7%)*  *1 (2.7%)*  *1 (2.7%)*  *1 (2.7%)*  *1 (2.7%)*  *1 (2.7%)*  *1 (2.7%)* |
| **Infections**   - Tuberculous lymphadenitis - Streptococcus pyogenes tonsillitis - Cutaneous infection - Viral infection   - HSV-1   - EBV   - CMV   - HIV   - Parotitis - Syphilis - Toxoplasmosis - Others | **64 (13.2%)**  *37 (57.8%)*  *3 (4.7%)*  *5 (7.8%)*  *6 (9.4%)*  *1 (1.6%)*  *2 (3.2%)*  *1 (1.6%)*  *1 (1.6%)*  *1 (1.6%)*  *1 (1.6%)*  *2 (3.2%)*  *9 (14.1%)* |
| **Inflammatory**   - Reactive vaccination - Hashimoto thyroiditis - Sarcoidosis - Castleman disease - Kikuchi-Fujimoto disease - Interferon treatment - Other | **32 (6.6%)**  *9 (28.1%)*  *5 (15.6%)*  *5 (15.6%)*  *1 (3.1%)*  *1 (3.1%)*  *1 (3.1%)*  *10 (31.3%)* |
| **Others**   - Branchial cyst - Thyroid nodules - Lipoma - Parotid tumour - Sialolithiasis - Fibroma - Vascular - Others | **76 (15.7%)**  *20 (26.3%)*  *12 (15.8%)*  *10 (13.2%)*  *7 (9.2%)*  *5 (6.6%)*  *3 (3.9%)*  *3 (3.9%)*  *16 (21.1%)* |

*Table S4: Epidemiological, clinical and analytical features according to the main etiological groups.*

|  | Unspecific  (n = 212) | Hematologic  (n = 64) | Oncologic  (n = 37) | Infectious  (n = 64) | Inflammatory  (n = 32) | Others  (n = 76) | *P* |
| --- | --- | --- | --- | --- | --- | --- | --- |
| *Epidemiology*  Age *(years)*  Gender *(women)*  Ethnicity   - Caucasian - Hispanic - Middle Eastern - African - Asian   Duration *(days)*  Drug consumption history  Oncological history  Risk factor  Sexual risk relation history  STD history  Recent immunization | 43.5 ± 1.2  127 (59.9%)  156 (73.6%)  29 (13.7%)  6 (2.8%)  7 (3.3%)  14 (6.6%)  90 (39-56)  65 (30.7%)  17 (8.0%)  37 (17.5%)  4 (1.9%)  3 (1.4%)  8 (3.8%) | 58.2 ± 2.2  24 (37.5%)  55 (85.9%)  5 (7.8%)  1 (1.6%)  1 (1.6%)  1 (1.6%)  60 (24-90)  24 (37.5%)  9 (14.1%)  5 (7.8%)  0  0  0 | 65.3 ± 2.0  8 (21.6%)  31 (83.8%)  2 (5.4%)  1 (2.7%)  2 (5.4%)  1 (2.7%)  34 (27-60)  26 (70.3%)  4 (10.8%)  7 (18.9%)  0  1 (2.7%)  1 (2.7%) | 39.9 ± 1.9  39 (60.9%)  23 (35.9%)  8 (12.5%)  13 (20.3%)  1 (1.6%)  19 (29.7%)  65 (30-248)  11 (17.2%)  5 (7.8%)  24 (37.5%)  5 (7.8%)  6 (9.4%)  1 (1.6%) | 42.5 (30.5-56.8)  23 (71.9%)  22 (68.8%)  3 (9.4%)  2 (6.3%)  1 (3.1%)  4 (12.6%)  136 ± 29  9 (28.1%)  2 (6.3%)  14 (43.8%)  0  0  10 (31.3%) | 49.9 ± 2.3  38 (50.0%)  57 (75.0%)  9 (11.8%)  5 (6.6%)  1 (1.3%)  4 (5.3%)  163 ± 26  29 (38.2%)  5 (6.6%)  14 (18.4%)  3 (3.9%)  3 (3.9%)  4 (5.3%) | **<0.001**  **<0.001**  **<0.001**  **0.001**  **<0.001**  0.660  **<0.001**  **0.033**  **0.008**  **<0.001** |
| *Features*  Indurated  Attached  Size *(mm)*  Fever  B symptoms  Toxic syndrome | 16/206 (7.8%)  7/208 (3.4%)  14.5 ± 0.6  4 (1.9%)  3 (1.4%)  6 (2.8%) | 35/56 (62.5%)  30/56 (53.6%)  32.5 (21.0-47.3)  4 (6.3%)  5 (7.8%)  12 (18.8%) | 26/35 (70.3%)  18/34 (48.6%)  36.2 ± 2.8  0  0  8 (21.6%) | 25/61 (41.0%)  12/60 (20.0%)  27.6 ± 2.0  11 (17.2%)  3 (4.7%)  4 (6.3%) | 1/27 (3.1%)  1/27 (3.1%)  15.8 ± 1.7  2 (6.3%)  1 (3.1%)  2 (6.3%) | 16/72 (22.2%)  14/72 (19.4%)  26.2 ± 1.6  2 (2.6%)  1 (1.3%)  1 (1.3%) | **<0.001**  **<0.001**  **<0.001**  **<0.001**  **0.026**  **<0.001** |
| *Distribution*  Cervical  Supraclavicular  Submandibular  Inguinal  Axillary  Mediastinum  Other  Extra-nodal  ≥2 non-contiguous territories | 123 (58.0%)  17 (8.0%)  39 (18.4%)  26 (14.5%)  18 (8.5%)  5 (2.4%)  8 (3.8%)  0  7 (3.3%) | 35 (54.7%)  26 (40.6%)  10 (15.6%)  24 (37.5%)  19 (29.7%)  11 (17.2%)  15 (23.4%)  14 (21.9%)  20 (31.3%) | 25 (67.5%)  12 (32.4%)  2 (5.4%)  2 (5.4%)  1 (2.7%)  6 (16.2%)  1 (2.7%)  4 (10.8)  3 (8.1%) | 29 (45.3%)  17 (26.6%)  7 (10.9%)  14 (21.9%)  5 (7.8%)  4 (6.3%)  4 (6.3%)  0  5 (7.8%) | 9 (28.1%)  8 (25.0%)  3 (9.4%)  5 (15.6%)  8 (25.0%)  2 (6.3%)  3 (9.4%)  1 (3.1%)  2 (6.3%) | 31 (40.8%)  10 (13.2%)  22 (28.9%)  7 (9.2%)  4 (5.3%)  0  3 (3.9%)  0  2 (2.6%) | **0.002**  **<0.001**  **0.013**  **<0.001**  **<0.001**  **<0.001**  **<0.001**  **<0.001**  **<0.001** |
| *Laboratory*  LDH (U/L)  β2-microglobulin *(mg/L)*  Hemoglobin *(g/dL)*  Leukocytes *(x10^3^ cells/μL)*  Neutrophils *(x10^3^ cells/μL)*  Lymphocytes *(x10^3^ cells/μL)*  ESR *(mm/h)* | 177 (150-206)  1.63 ± 0.07  14.2 ± 0.1  7.30 ± 0.14  3.77 (3.00-4.60)  2.48 (1.90-3.00)  15 (6-29) | 193 (169-295)  2.17 (1.67-3.28)  13.3 ± 0.2  9.08 ± 0.68  5.76 ± 0.59  2.22 ± 0.22  18 (6-44) | 193 (149-244)  1.80 ± 0.09  13.8 ± 0.3  9.10 (7.26-10.60)  5.68 ± 0.33  2.16 ± 0.19  10 (5-15) | 170 (155-183)  1.99 ± 0.14  13.8 ± 0.2  7.39 ± 0.30  3.70 (2.78-4.88)  2.15 ± 0.09  32 ± 6 | 183 (156-235)  2.13 ± 0.26  13.7 ± 0.3  8.32 ± 0.50  4.96 ± 0.35  2.27 ± 0.17  29 ± 6 | 192 ± 10  1.66 ± 0.10  14.2 ± 0.2  8.38 ± 0.31  4.88 ± 0.24  2.51 ± 0.10  16 ± 2 | **0.002**  **<0.001**  **0.002**  **<0.001**  **0.044**  0.090  0.056 |

*Categorical variables were expressed as percentages, and continuous variables by mean ± standard error of the mean, or median (IQR), according to their normal distribution.* *ESR = erythrocyte sedimentation rate; LDH = Lactate dehydrogenase; STD = Sexually transmitted diseases.*

| Table S5: Percentage of malignancy according to LA distribution and size. | | | | | | |
| --- | --- | --- | --- | --- | --- | --- |
| **Distribution** | **Cervical** | **Submandibular** | **Supraclavicular** | **Inguinal** | **Axillary** | **Mediastinum** |
| **N**  **Malignancy** | 252  23.8% | 83  14.5% | 90  42.2% | 87  29.9% | 55  36.4% | 28  60.7% |
| **LA > 10mm**  **Malignancy** | 171/236  33.3% | 59/79  18.6% | 70/83  50% | 67/82  34.3% | 39/51  48.7% | 26/28  65.4% |
| **LA > 20mm**  **Malignancy** | 103/236  42.7% | 31/79  29% | 45/83  57.8% | 39/82  51.3% | 20/51  70% | 16/28  81.3% |
| **LA > 25.5mm**  **Malignancy** | 74/236  48.6% | 24/79  33.3% | 38/83  60.5% | 32/82  56.3% | 12/51  80% | 12/28  83.3% |
|  | | | | | | |

*Table S6: Independent clinical predictors of malignancy in patients with unexplained lymphadenopathy (LA)*

| **Factor** | **Malignant LA**  **(N = 101)** | **Non-malignant LA**  **(N = 384)** | **Multivariable Analysis** | | |
| --- | --- | --- | --- | --- | --- |
|  |  |  | **OR** | **95% CI** | **P** |
| Age (per 10-year increase)  Size (per 5-mm increase)  Gender (men)  Supraclavicular location  Indurated consistency | 60.7 ± 1.6  33.5 (21.8-48.0)  69/101 (68.3%)  38/101 (37.6%)  61/91 (67.0%) | 42 (30 – 57)  16.5 (10.0-25.0)  171/384 (44.5%)  52/384 (13.5%)  58/366 (12.8%) | 1.71  1.36  3.25  4.96  3.42 | 1.41 – 2.07  1.21 – 1.53  1.66 – 6.37  2.46 – 9.99  1.78 – 6.60 | <0.001  <0.001  0.001  <0.001  <0.001 |

Model performance: AUC = 0.91

Supplementary Figures:

Figure S4: Flow diagram of patient inclusion:


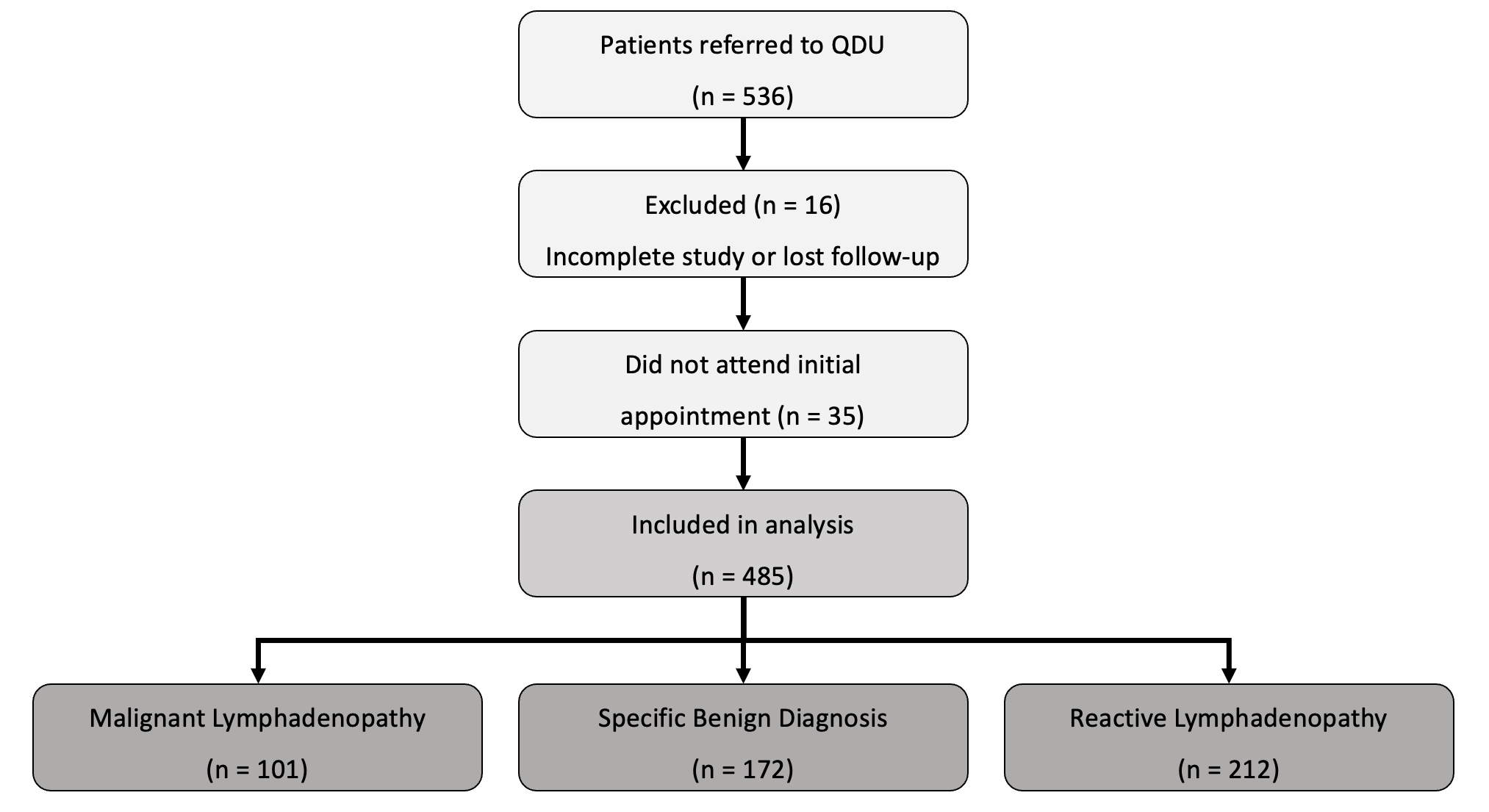


Figure S5: Age-frequency histogram according to malignant or benign aetiology of LA*.*

**Malignant**

NO YES

x̄ = 44.1

sem = 0.9

x̄ = 60.7

sem = 1.6


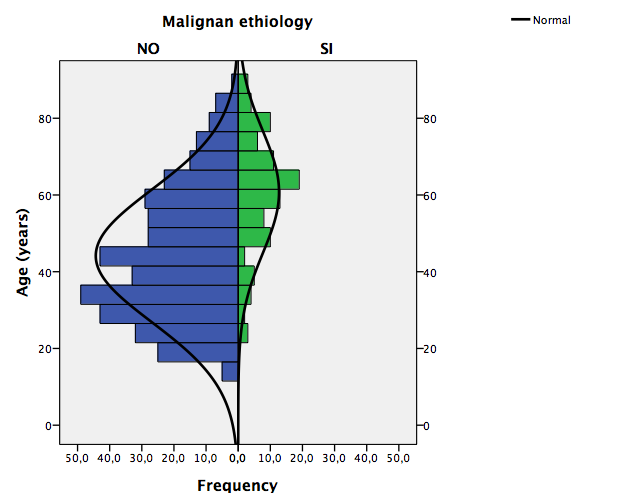


*p <0.001*

*Variables were expressed by mean (*x̄*) ± standard error of the mean (sem).*
